# A rapid α‐synuclein seed assay of Parkinson’s disease CSF panel shows high diagnostic accuracy

**DOI:** 10.1002/acn3.51280

**Published:** 2020-12-29

**Authors:** Christina D. Orrù, Thong C. Ma, Andrew G. Hughson, Bradley R. Groveman, Ankit Srivastava, Douglas Galasko, Rachel Angers, Patrick Downey, Karen Crawford, Samantha J. Hutten, Un Jung Kang, Byron Caughey

**Affiliations:** ^1^ Laboratory of Persistent Viral Diseases Rocky Mountain Laboratories National Institute of Allergy and Infectious Diseases NIH Hamilton Montana; ^2^ Department of Neurology New York University Grossman School of Medicine New York New York; ^3^ Department of Neurosciences University of California‐San Diego La Jolla California; ^4^ UCB Biopharma SRL Braine l'Alleud Belgium; ^5^ Laboratory of Neuro Imaging Mark and Mary Stevens Neuroimaging and Informatics Institute Keck School of Medicine of USC University of Southern California Los Angeles California; ^6^ Michael J. Fox Foundation New York New York

## Abstract

**Background:**

Assays that specifically measure α‐synuclein seeding activity in biological fluids could revolutionize the diagnosis of Parkinson’s disease. Recent improvements in α‐synuclein real‐time quaking‐induced conversion assays of cerebrospinal fluid have dramatically reduced reaction times from 5‐13 days down to 1‐2 days.

**Objective:**

To test our improved assay against a panel of cerebrospinal fluid specimens from patients with Parkinson’s disease and healthy controls from the MJ Fox Foundation/NINDS BioFIND collection.

**Methods:**

Specimens collected from healthy controls and patients with clinically typical moderate‐to‐advanced Parkinson’s disease were tested without prior knowledge of disease status. Correlative analyses between assay parameters and clinical measures were performed by an independent investigator.

**Results:**

BioFIND samples gave positive signals in 105/108 (97%) Parkinson’s disease cases versus 11/85 (13%) healthy controls. Receiver operating characteristic analyses of diagnosis of cases versus healthy controls gave areas under the curve of 95%. Beyond binary positive/negative determinations, only weak correlations were observed between various assay response parameters and Parkinson’s disease clinical measures or other cerebrospinal fluid analytes. Of note, REM sleep behavioral disorder questionnaire scores correlated with the reaction times needed to reach 50% maximum fluorescence. Maximum fluorescence was inversely correlated with Unified Parkinson's Disease Rating Scale motor scores, which was driven by the patients without REM sleep behavioral disorder.

**Conclusions:**

Our improved α‐synuclein seed amplification assay dramatically reduces the time needed to diagnose Parkinson’s disease while maintaining the high‐performance standards associated with previous α‐synuclein seed assays, supporting the clinical utility of this assay for Parkinson’s disease diagnosis.

## Introduction

The accumulation of aggregates of the protein α‐synuclein (αSyn) characterizes neurodegenerative diseases collectively termed α‐synucleinopathies, which include Parkinson’s disease (PD), Parkinson’s disease with dementia (PDD), dementia with Lewy bodies (DLB), and multiple system atrophy (MSA), as well as prodromal stages manifesting as REM sleep behavioral disorders (RBD).^1^ The ability to diagnose these disorders early and accurately will likely be critical in the development and successful application of disease‐modifying therapies. However, currently, these diseases can be difficult to diagnose and discriminate from other neurodegenerative disorders in living patients. For example, the accuracy of clinically based diagnosis of PD is complicated by phenotypic heterogeneity that can overlap with several other neurodegenerative disorders.^2^, ^3^, ^4^, ^5^, ^6^ Imaging techniques can detect deficits in dopaminergic function associated with parkinsonism, but do not discriminate PD from other disorders involving the loss of dopaminergic signaling such as progressive supranuclear palsy and other tauopathies.^7^ Biochemical analyses of accessible biospecimens have identified factors with significantly different mean concentrations between PD and control populations but with overlapping ranges that compromise their utility in diagnosing individuals.^8^, ^9^

Recently, αSyn seeding activity has been identified as a biomarker with high diagnostic utility because it differs more markedly between patients with and without synucleinopathy in brain, cerebrospinal fluid (CSF),^10^, ^11^, ^12^, ^13^, ^14^, ^15^, ^16^ nasal brushings,^17^ and skin.^18^ This marker has been detected using ultrasensitive seeded aggregation assays, that is, αSyn real‐time quaking‐induced conversion (αSyn RT‐QuIC)^10^, ^12^, ^13^, ^15^, ^17^, ^19^ and protein misfolding cyclic amplification (αSyn PMCA).^11^, ^16^ These assays are based on the ability of disease‐associated αSyn (αSyn^D^), which is often fibrillar, to propagate by seeding the recruitment of nonfibrillar αSyn into the growing assembly. Samples containing αSyn^D^ seeds are mixed with a vast stoichiometric excess of soluble recombinant αSyn and, over time, recombinant αSyn amyloid fibrils accumulate that are detected by an increase in the fluorescence of thioflavin T (ThT). This seeded polymerization mechanism can amplify the presence of αSyn^D^ seeds in a biospecimen by a billion‐fold or more, allowing detection of as little as 100 ag of seed.^12^

Analyses of CSF specimen panels using αSyn PMCA and first‐generation αSyn RT‐QuIC assay (hereafter αSyn RT‐QuIC‐FG) have yielded high diagnostic performance for distinguishing PD versus clinical controls with greater than 80% specificity (percentage of non‐PD cases giving a negative response) and 85% sensitivity (percentage of PD cases giving a positive response) depending on assay implementation and patient cohort.^10^, ^11^, ^12^, ^13^, ^14^, ^15^, ^16^ A comparison of αSyn RT‐QuIC‐FG and PMCA assays using the same CSF samples from the Fox Investigation for New Discovery of Biomarkers (BioFIND) panel containing PD patients (n = 105) and healthy controls (HC, n = 79) showed 92% concordance with 93% specificity and 97% sensitivity for concordant samples.^14^ One practical shortcoming of these first‐generation αSyn seed amplification assays is the assay duration, requiring 5‐13 days to obtain the final results.^10^, ^11^ However, a more rapid αSyn RT‐QuIC requiring only 1‐2 days (hereafter called αSyn RT‐QuICR) has been shown with initial CSF test panels to have comparable diagnostic sensitivity and specificity.^12^, ^15^, ^19^ A combination of factors allowed for the greater rapidity of RT‐QuICR, including αSyn substrate purification method, higher reaction temperature, beads, and sodium dodecyl sulfate (SDS). The use of a K23Q mutant αSyn substrate also provided modest improvements compared to wildtype αSyn in the initial study.^12^

Here, we report blinded testing of the Michael J. Fox Foundation/NINDS BioFIND CSF samples from patients with moderate to severe PD and healthy controls using the αSyn RT‐QuICR assay.

## Patients and Methods

### Participants

Study protocols were approved by the institutional review board of the individual sites as outlined in the initial BioFIND description.^20^ Clinical data and biosamples used in preparation of this article were obtained from the BioFIND database (http://biofind.loni.usc.edu/). For up‐to‐date information on the study, visit michaeljfox.org/biospecimens. Testing of the specimens at NIAID was performed under NIH OHSRP Exemption #18‐NIAID‐00525. Participants in the study had moderate‐to‐advanced PD according to the United Kingdom PD Society Brain Bank clinical diagnostic criteria and represented all Hoehn and Yahr stages. HC subjects were enrolled at eight movement disorders centers in the United States. Convenience sampling was carried out on those who volunteered for the study without randomization or consecutive enrollment. Additional inclusion criteria for PD patients have been described^20^ and included: motor signs such as bradykinesia, rigidity, and resting tremor; disease duration ≥5 years and onset at 50‐75 years. Furthermore, PD patients were recruited if they had a well‐established response to dopaminergic agents and/or amantadine. Additionally, subjects were excluded when they had atypical or secondary parkinsonian syndromes; history of deep brain stimulation or ablative brain surgery; a history of cancer within 5 years preceding enrollment; autoimmune, liver, or hematological disorders; or any condition precluding lumbar puncture. HCs were sex and age matched to PD subjects, had no history of neurological disorders, and scored ≥26 on the Montreal Cognitive assessment (MoCA). Control subjects with any first‐degree family member with PD were excluded to reduce the chance of enrolling prodromal cases, and other exclusion criteria for HCs were similar to those for PD subjects.

### CSF samples

Clinical evaluation and biospecimen collection were performed during two visits, as described in detail previously.^20^ Briefly, CSF was collected in 15 mL conical polypropylene tubes and centrifuged at 2000*g* for 10 minutes at room temperature. One milliliter aliquots of the supernatant were transferred to 2 ml polypropylene tubes, and immediately frozen on dry ice and/or directly transferred into a –80°C freezer for storage. Samples were then shipped to the biorepository on dry ice at a later date. The repository further aliquoted the CSF into 200 µL portions for distribution. CSF αSyn, phospho‐tau, and tau were measured previously and are part of the BioFIND data repository. αSyn was quantitated by ELISA (BioLegend; cat. 844101, San Diego, CA). Phospho‐tau and tau were quantitated simultaneously along with Aβ1‐42 using a highly standardized microbead‐based research‐use‐only immunoassay (Fujirebio; Alz Bio3 kit, Ghent, Belgium).^21^

### K23Q recombinant α‐Syn purification

The K23Q mutation of the αSyn sequence (Accession No. NM_000345.3) was engineered using Q5 Site‐Directed Mutagenesis (NEB) using the primers CCACACCCTGTTGGGTTTTCTCAG and CAGAAGCAGCAGGAAAGAC, as previously described,^12^ using a pET28 vector with an N‐terminal His‐tag (EMD Biosciences). The plasmid was transformed into BL21(DE3) Escherichia coli (EMD Biosciences). K23Q recombinant αSyn was purified as previously described.^22^

### αSyn RT‐QuICR

RT‐QuIC reactions were carried out in black 96‐well plates with a clear bottom (Nalgene Nunc International). Each well was preloaded with six glass beads (0.8 mm in diameter, OPS Diagnostics). Quadruplicate reactions were seeded with 15 μL of CSF. Each RT‐QuIC reaction mix (prior to addition of CSF) was 85 μL of solution^12^ adjusted to give final reaction concentrations of 40 mM sodium phosphate buffer (from a stock solution of 0.5 M monobasic and 0.5 M dibasic sodium phosphate solutions mixed at a ratio of 1:19 for a pH 8.0), 170 mM NaCl, 0.1 mg/mL K23Q recombinant αSyn (filtered through a 100 kD MWCO filter immediately prior to use), 10 μM thioflavin T (ThT) and 0.0015% SDS. The plates were closed with a plate sealer film (Nalgene Nunc International) and incubated at 42°C in a BMG FLUOstar Omega plate reader. During the incubation plates were subjected to cycles of 1 min shaking (400 rpm double orbital) and 1 min rest for at least 48 h. ThT fluorescence measurements (450 +/− 10 nm excitation and 480 +/− 10 nm emission; bottom read) were taken every 45 min with fluorimeter gain settings adjusted to maintain fluorescence responses within an unsaturated range (in most cases). The fluorescence threshold for a positive result was calculated as 10% of the maximum value any well reached per experiment. The threshold was calculated individually for each 96‐well plate to account for differences across experiments and between fluorescent plate readers. For a sample to meet the criteria for being considered positive, the fluorescent signal exceeded the threshold in at least 50% of the replicate wells (e.g., ≥2 of 4) prior to 48 hours.

Other parameters of αSyn seeding kinetics were also calculated from the ThT fluorescence curves including: T50, the time for a reaction to reach 50% of the maximum fluorescence of that curve; lag, the reaction time required to cross the fluorescence threshold; and AUC‐fluor, the area under the ThT fluorescence curve integrated over the assay duration. Assays with subthreshold ThT signals were artificially assigned T50 and lag values of 50 hours, that is, the maximum assay duration.

### HC and PD CSF pool for RT‐QuICR quality control

Individual CSF samples from either nonsynucleinopathy patients or patients with a definite diagnosis of Parkinson’s (NIH NeuroBioBank) were combined to create HC and PD pools, respectively. These pools were used as controls, with each plate including quadruplicate reactions seeded with the HC and PD pool.

### Statistical analysis

Differences between cases and controls were compared using percent for categorical data and median and range for continuous data. Categorical data were assessed using the Chi‐squared test, and differences in continuous data were assessed using the Mann–Whitney test. Sensitivity, specificity, negative predictive value, and positive predictive value were calculated for comparisons between clinical diagnosis and positive assays determined from maximum fluorescence or from continuous values of other assay parameters. Correlations were calculated for the assay results between the techniques as well as between the assay results and clinical characteristics. Logistic regression was used to generate receiver operating characteristic (ROC) curves to assess the relationship between assay results and clinical diagnosis of PD. We chose the optimal cutoff by selecting the value at which the sum of sensitivity and specificity was maximal. A two‐tailed alpha of 0.05 was used. ROC data were calculated using the ModelGood package in R. Other data were analyzed and plotted with GraphPad Prism 8.4.

### Data availability statement

De‐identified participant data, which includes clinical phenotypes, demographics, and assay results, are available online (http://biofind.loni.usc.edu/) upon registration with the Michael J. Fox Foundation for Parkinson’s Research. The study protocol is also available at: https://www.michaeljfox.org/news/biofind. The statistical analysis is available upon request from the authors.

### Ethical approval and patient consent

All study protocols and recruitment strategies were approved by the Institutional Review Boards (IRBs) for the University of Rochester Clinical Trials Coordination Center (CTCC) and individual sites. Subjects provided written informed consent before participation in the study (ClinicalTrials.gov NCT01705327).

We confirm that we have read the Journal’s position on issues involved in ethical publication and affirm that this work is consistent with those guidelines.

## Results

**Figure 1:**
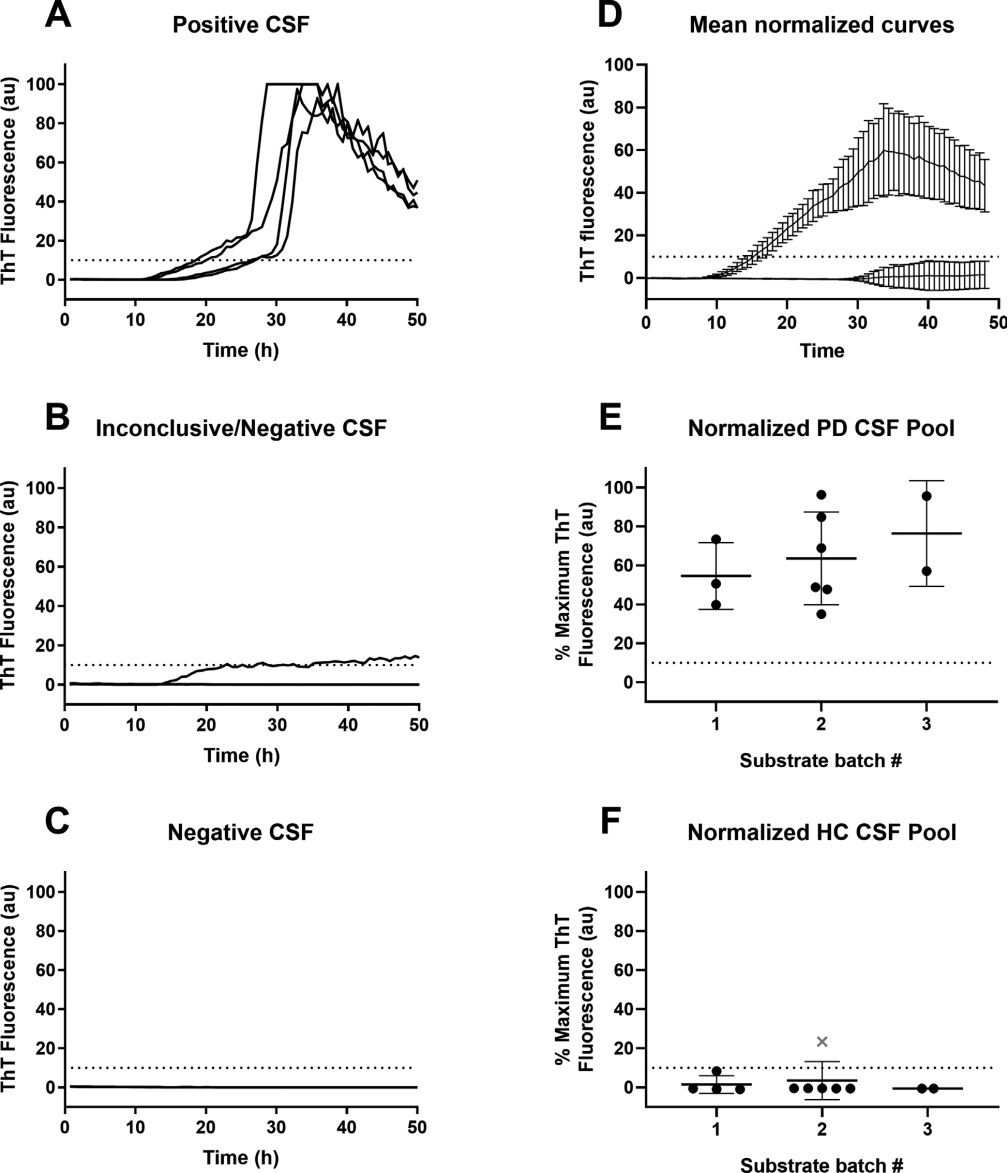
1Representative blinded αSyn RT‐QuICR testing of individual CSFs and ThT fluorescence for HC and PD CSF standards. Panels A‐C. Fluorescence (in arbitrary units, au) for each replicate well seeded with 15 µL of CSF, normalized to the highest fluorescence reading for any well on the plate. Panel D: Average % maximum ThT fluorescence (normalized to highest well on the plate) over time for reactions seeded with 15 µL of HC or PD CSF pools. Panels E and F: % maximal ThT fluorescence for reactions seeded with HC or PD CSF pools using batches 1‐3 of K23Q rec‐αSyn substrate. Data points show mean ± SD of quadruplicate wells normalized to the highest ThT value for a well on the same plate. The dotted line indicates fluorescence positivity threshold. “x” designates the reading from a sample that did not meet overall criteria for being a positive despite having an above‐threshold fluorescence response crossing threshold because only one of four wells was positive.

Each sample was assayed in quadruplicate in 96‐well plates with the operator blinded to sample identity using 15 µL CSF per reaction as described.^12^ The ThT fluorescence responses for positive and negative results (Figure 1) were consistent with our previous results.^12^ Single well traces in Figure 1 show representative results for CSF samples deemed to be positive (Figure 1A), inconclusive/negative (Figure 1B), and a negative (Figure 1C). Inconclusive/negative tests, that is, those with one positive of four replicate reactions, were repeated. If the sample then was positive in 0‐1 of four replicate reactions, it was considered negative overall. If it gave ≥ 2 positive of four replicate reactions it was considered positive overall.

Each plate also included quadruplicate reactions seeded with CSF pooled from nonsynucleinopathy (HC) or PD patients, as negative and positive controls, respectively (see Methods). Reactions seeded with the HC CSF pool showed fluorescence below our calculated threshold (Figure 1D, dotted line), with only one instance in which one of 4 replicate wells was positive. Even in this case, the HC pool sample was deemed negative overall in this experiment as it did not meet the sample positivity criterion of having at least 50% positive wells.^12^ Positive control reactions seeded with the PD CSF pool showed increases in ThT fluorescence starting between 8 and 12 h with all four replicate reactions exceeding the positivity threshold (Figure 1D,E), whereas the reactions seeded with the HC CSF pool were consistently below threshold (Figure 1D,F). The mean maximum normalized ThT fluorescence values obtained in PD CSF‐seeded reactions using three different K23Q recombinant αSyn substrate batches were not significantly different (Figure 1E).

The BioFIND CSF sample set that we tested included 108 clinically diagnosed cases of PD and 85 healthy controls which showed no clinical signs of neurological disease. All but 3 PD and six controls were the same as those tested in the previous study using the αSyn RT‐QuIC‐FG and PMCA.^14^ Demographics of the cohort providing those samples were reported previously.^14^, ^20^ The demographics of the entire cohort for this study are summarized in Supplementary Table S1.

Using our binary decision criteria based on maximum fluorescence, RT‐QuICR testing of the BioFIND CSF samples yielded positive assays for 105/108 PD samples and 11/85 healthy controls, resulting in a diagnostic sensitivity (percentage of PD cases giving a positive response) of 97% (95% CI: 91‐99%) and specificity (percentage of PD cases giving a positive response) of 87% (CI: 78‐93%) (Table 1). This was comparable to previous results with αSyn RT‐QuIC‐FG and PMCA with BioFIND samples.^14^

**Table 1:** Diagnostic performance of αSyn seeding aggregation assays on the BioFIND cohort using maximum fluorescence values.

	RT‐QuICR (Caughey)	PMCA (Soto)^1^	RT‐QuIC‐FG (Green)^1^	Concordant^2^
Sensitivity	97.2% (92.1‐99.4)	95.2% (90.6‐98.0)	96.2% (91.4‐98.7)	98.0% (93.1‐99.8)
Specificity	87.1% (78.0‐93.4)	89.9% (83.8‐93.5)	82.3% (76.0‐85.6)	92.3% (83.0‐97.5)
PPV	90.5% (83.7‐95.2)	92.6% (88.1‐95.2)	87.8% (83.5‐90.1)	95.2% (89.2‐98.4)
NPV	96.1% (89.0‐99.2)	93.4% (87.1‐97.2)	94.2% (87.0‐98.0)	96.8% (88.8‐96.6)
AUC (ROC)	92.14%	92.56%	89.23%	95.17%

Abbreviations: AUC (ROC), area under the receiver operator characteristics analysis curve using maximum fluorescence data; NPV, negative predictive value; PPV, positive predictive value.

Ranges in parentheses are 95% confidence intervals.

^1^ PMCA and RT‐QuIC‐FG results were obtained from the BioFIND data repository, which were deposited from a previous study (ref 14).

^2^ Calculated from concordant results across all three assays which include 102 PD and 65 HC subjects.

**Figure 2:**
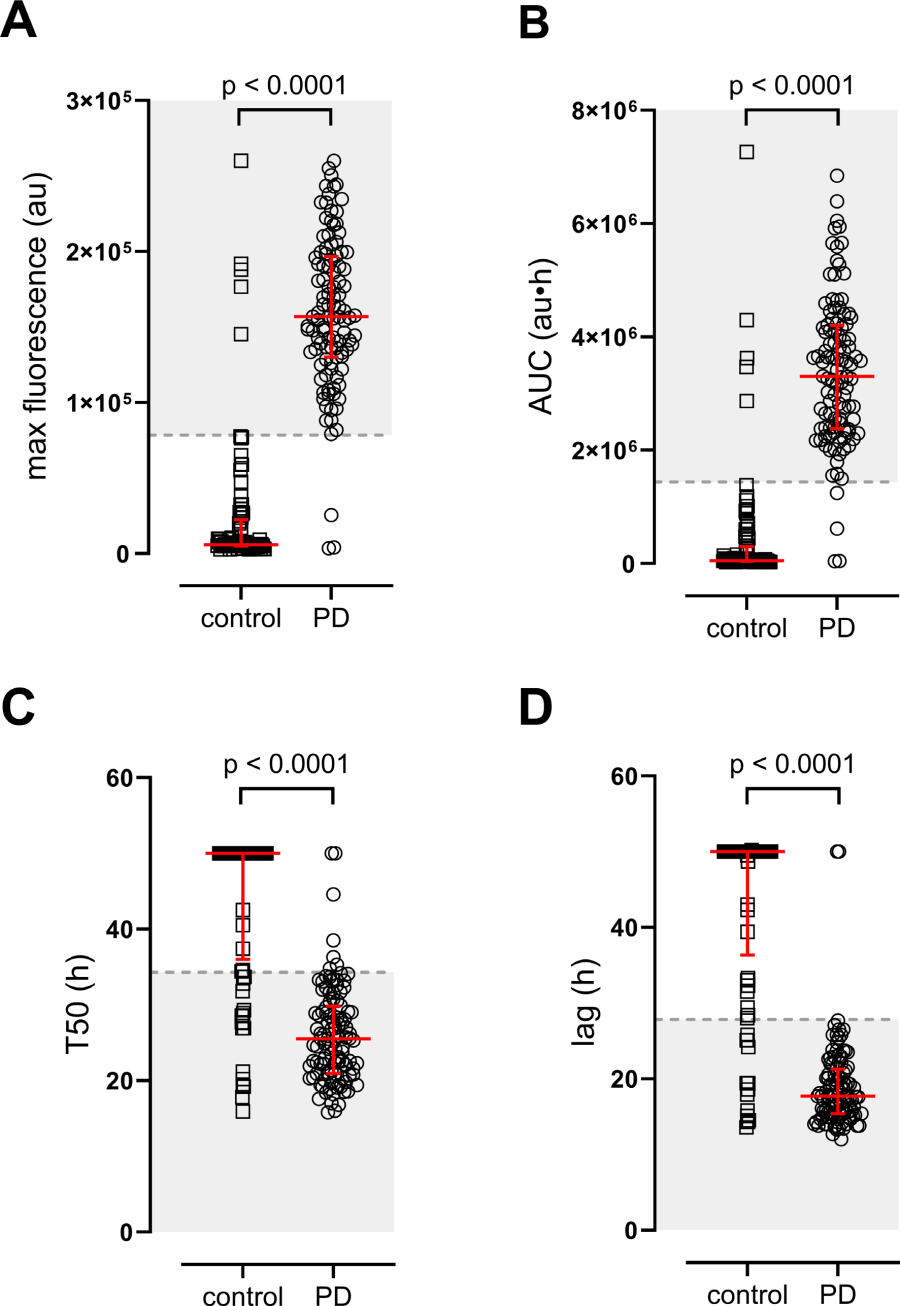
2RT‐QuICR assay parameters of healthy control and PD patients. Dot plots of A) Maximum ThT fluorescence in relative arbitrary units (au), B) area under the fluorescence curve (i.e., AUC‐fluor; au·h), C) time needed to reach 50% of maximum fluorescence (T50), and D) time needed to reach threshold fluorescence (lag) are shown for each subject. Each symbol represents the average of quadruplicate assay results. Thick black bars are due to the overlap of multiple data points. The red line and error bars represent the median and interquartile range of each group. The dashed line represents the cutoff value and the shaded area represents the values that constitute a positive result based on ROC analysis (see Table 2). For T50 and lag measures, a maximum time of 50 h was assigned for samples that had no amplification. P‐values of two‐tailed Mann–Whitney tests are reported, Control: n = 85; PD: n = 108.

We also evaluated the diagnostic performance of other parameters derived from RT‐QuICR amplification curves, including the maximum ThT fluorescence, area under curve of fluorescence signal (AUC‐fluor), lag time (time to threshold), and time to half‐maximum fluorescence (T50). Using receiver operator characteristics (ROC) analyses of these parameters, we selected cutoff values for each measure that maximized the combined specificity and sensitivity for PD diagnosis (Figure 2 and Table 2). Maximum fluorescence and AUC‐fluor were the best able to discriminate PD from healthy controls with virtually equal performance, giving >94% values for area under ROC curves AUC (ROC). Of note, using the ROC‐derived threshold for maximum fluorescence improved the specificity of our assay compared to the per‐plate criteria that was used to assign assay positivity (from 87.1% to 94.1%; Table 1 and Table 2). All parameters were able to discriminate PD from health controls with AUC (ROC) values ranging from 88.99‐94.87% (Table 2).

**Table 2:** ROC analysis of individual RT‐QuICR assay parameters for PD diagnosis.

	Sensitivity	Specificity	PPV	NPV	AUC (ROC)	Threshold
Max Fluor	97.2% (92.1‐99.4)	94.1% (86.8‐98.1)	99.5% (89.7‐98.5)	96.4% (89.8‐99.2)	94.28%	>78462 au
AUC‐fluor	96.3% (90.8‐99.0)	94.1% (86.8‐98.1)	95.4% (89.6‐98.5)	95.2% (88.3‐98.7)	94.87%	>1441404 au⋅h
T50	93.5% (87.1‐97.4)	78.8% (68.6‐86.9)	84.9% (77.2‐90.8)	90.5% (81.5‐96.1)	88.99%	<34.30 h
Lag	98.1% (93.5‐99.8)	83.5% (73.9‐90.7)	88.3% (81.2‐93.5)	97.3% (90.5‐99.7)	91.55%	<27.85 h

Ranges in parentheses are 95% confidence intervals.

Abbreviations: au, arbitrary units; AUC, area under curve; AUC‐fluor, AUC of fluorescence versus time plot; Lag, reaction time to threshold; Max Fluor, maximum fluorescence; NPV, negative predictive value; PPV, positive predictive value; ROC, receiver operator characteristics; T50, reaction time to reach half‐maximal fluorescence.

A previous study comparing α‐Syn RT‐QuIC‐FG and PMCA assays using the same BioFIND cohort showed high specificity and sensitivity similar to our assay, though no correlation was found between assay parameters and clinical measures of PD severity.^14^ As our RT‐QuICR differs from these other assays in some key areas, we first tested whether maximum fluorescence or T50 values correlated for the CSF samples that were tested in all three assays (control: n = 79, PD n = 105). Neither maximum fluorescence nor T50 values correlated between our assay and the PMCA assay as implemented by the Claudio Soto laboratory (Supplementary Figure S1). For the RT‐QuIC‐FG assay from the Alison Green laboratory, there was a weak but statistically significant correlation of T50 times (Supplementary Figure S1B); no comparison of maximum fluorescence could be made due to the nature of the RT‐QuIC‐FG data (MJFF BioFIND data repository). Together, these findings suggest that each assay may reflect specific aspects of αSyn seeded aggregation.

**Figure 3:**
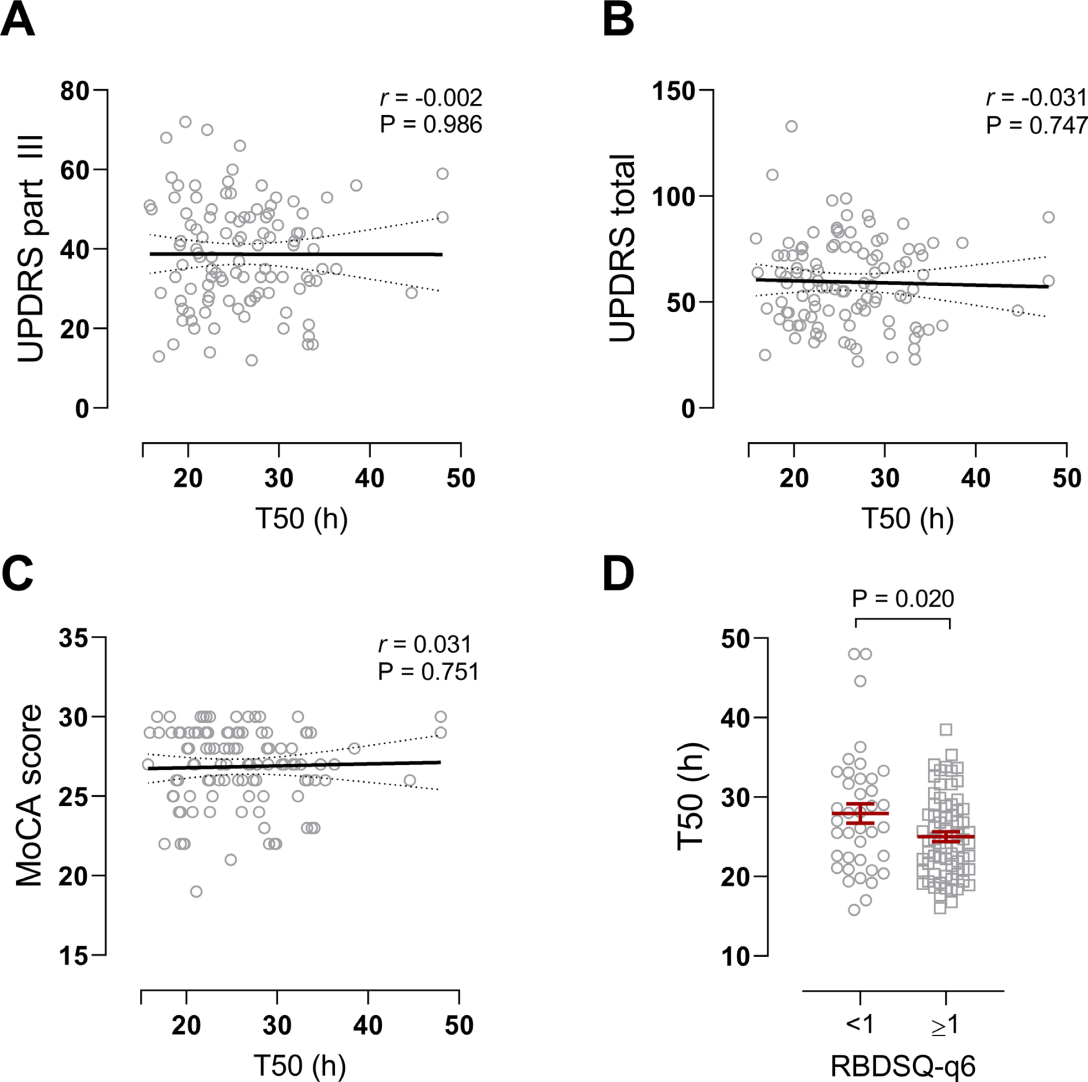
Correlation between T50 values and clinical measures. Scatter plots of A) UPDRS‐III and B) UPDRS total while off PD medication, and C) MoCA scores versus T50 times for PD patients. No correlations were evident between these clinical measures and T50. Pearson’s r and p‐values are reported, n = 108. D) T50 values of PD patients dichotomized by RBD questionnaire – question #6 (RBDSQ‐q6) <1 and ≥ 1 to identify “probable RBD.” p = 0.020, two‐tailed Student’s t‐test, <1 group: n = 39; ≥1 group: n = 69.

Unlike the previous αSyn RT‐QuIC‐FG and PMCA assays of the same BioFIND cohorts, some weak correlations between our RT‐QuICR maximum fluorescence and T50 values and the clinical and biochemical measures were evident (Supplementary Table S2). For instance, T50 and REM sleep behavioral disorder (RBD) questionnaire scores were inversely correlated, indicating that lower T50 values are associated with RBD symptoms. However, T50 values had no correlation with UPDRS‐total, UPDRS‐III (motor), or MoCA (cognitive) scores (Figure 3A‐C). We stratified PD patients based on RBD‐Screening Questionnaire (RBDSQ) question 6 ≥1 as having probable RBD,^23^ as using a single item to assess dream enactment has better discriminatory power for RBD diagnosis,^23^, ^24^, ^25^ and found that those experiencing RBD symptoms had lower T50 scores at the population level (Figure 3D), whereas the maximum fluorescence did not correlate with RBD scores (Supplementary Table S2). Using these criteria, we found that the weak inverse correlation between maximum fluorescence and UPDRS‐III scores and improvement in UPDRS‐III while on PD medication was driven by the RBD‐negative group (Supplementary Table S2, Supplementary Figure S2). Together, these data indicate that assay kinetics of the RBD‐positive and RBD‐negative groups differ and suggest that some characteristics of αSyn seeding species or CSF constituents may be different in these two groups. Additionally, T50 times showed a positive correlation with CSF phosphorylated tau (Thr181) levels, whereas maximum fluorescence showed an inverse correlation with CSF αSyn and total tau levels (Supplementary Table S2).

## Discussion

Our study using the BioFIND CSF sample set reaffirms the already strong evidence of the high diagnostic sensitivity and accuracy of αSyn seeding assays for the diagnosis of Parkinson’s disease. Moreover, our RT‐QuICR has significantly improved the time needed to complete the assay without sacrificing assay performance, therefore enhancing the practicality of the RT‐QuICR over the previous assays that have been tested with this sample set.^14^ Given the lack of diagnostic biomarkers for PD with high enough discriminatory power, these αSyn seeding assays are poised to dramatically improve the diagnosis of PD. As αSyn misfolding and aggregation are key processes to PD pathogenesis, understanding how αSyn seeding assays inform on the nature and quantity of seeding species will provide insight on how αSyn contributes to the disease process.

The BioFIND PD patient cohort exclusively enrolled patients with moderate to severe disease and specifically excluded patients with significant histories of dementia. Patients were evaluated at an initial visit and samples were collected 2 weeks later^20^ without long‐term follow‐up. Therefore, one cannot exclude the possibility that some of the apparent false‐positive results that we obtained could be from cases of early PD or other synucleinopathies that were not observed clinically at the time. This is consistent with two previous reports using RT‐QuICR that have identified early PD cases, and even idiopathic REM sleep behavioral disorder cases that eventually progressed to PD.^12^, ^15^ Five of the 11 apparent false positives and two of the three false negatives that we observed here were also indicated previously as such by αSyn PMCA and αSyn RT‐QuIC‐FG.^14^ The fact that all three assays appear to have misidentified a small subset of samples suggests either that these cases were initially misdiagnosed or that the samples contained an unidentified component that influenced the outcome of all three tests.^26^ Experience with a larger number of subjects will be needed to clarify this issue.

As was the case with the previous published assays of the BioFIND cohort,^14^ there was no strong correlation between individual RT‐QuICR assay parameters, including maximum fluorescence, T50, lag time, and AUC‐fluor.^14^ However, we observed that the “probable RBD” group showed significantly shorter T50 times than those with negative responses in the RBD questionnaire. Interestingly, weak inverse correlations between maximum fluorescence and clinical features such as motor deficit (MDS‐UPDRS‐III) and improvement of motor deficit with PD medications were noted only in those without RBD. Although higher concentrations of recombinant oligomeric αSyn seeds shortens T50 and increases maximum fluorescence,^12^ differences in the αSyn strains could alter aggregation kinetics and maximum fluorescence.^16^ Shorter T50 or higher maximum fluorescence was associated with lower CSF phosphorylated tau, total tau, and αSyn. Lower levels of these proteins were noted in PD subjects compared to controls in a previous study.^27^ However, whether these proteins or other components of the biospecimen matrix (such as CSF) influence the kinetic parameters needs further study. Therefore, interpretation of these intriguing differences in assay kinetics will need more nuanced understanding of the determinants αSyn aggregation.

In summary, our RT‐QuICR assay significantly decreases the run time of αSyn^D^ seeded aggregation assays, while maintaining equal diagnostic performance to RT‐QuIC‐FG and PMCA assays in identifying PD patients in the BioFIND cohort.^14^ The high diagnostic accuracy of these assays is well validated in different cohorts and by three different assays from independent laboratories in blinded fashion, raising the possibility of their use as an individual diagnostic test. This is in contrast to most current biomarkers that are limited to population‐level discrimination of PD. At the current state of the art, seed amplification assays such as our improved RT‐QuICR assay represent powerful tools for the diagnosis of PD. However, whether or not such assays could be used to monitor the status and progression of PD will require further understanding of how the molecular and pathogenic processes that cause PD relate to assay parameters. Additional studies with sample sets from well‐characterized patients of wider ranges of PD stages including prodromal stages, preferably with data from long‐term clinical follow‐up and neuropathological examination, are needed.

## Conflict of Interest

CDO, AGH, BRG, and BC have a patent Provisional (US): 62/567,079 pending, a patent PCT: PCT/US2018/052968 pending, a patent Canada: 3074914 pending, a patent Europe: 18786583.7 pending, and a patent U.S.: 16/652,804 pending. TCM, AS, SH, and KC report no relevant conflicts of interest. DG is a Consultant for Amprion, Inc. and receives research funding from the Michael J. Fox Foundation. RA and PD are full‐time employees of UCB Biopharma. UJK was supported by Michael J Fox Foundation for this study and is on the Scientific Advisory Board of Amprion, Inc. BioFIND is sponsored by The Michael J. Fox Foundation for Parkinson’s Research (MJFF) with support from the National Institute of Neurological Disorders and Stroke (NINDS).

## Authors’ Contributions

CDO, UJK, BC, DG, SH, TCM, RA, and PD involved in conception. CDO, AGH, BRG, AS, and KC carried out execution of experiments. TCM, UJK, and BRG involved in data comparisons and statistical analyses. BC, CDO, BRG, UJK, and TCM involved in writing the first draft of the article. All authors involved in editing the draft.

## Supporting information


**Figure S1.** Comparison of RT‐QuICR, RT‐QuIC‐FG, and PMCA αSyn seeding assays. Scatter plots of A) maximum fluorescence in arbitrary units (au) and B) T50 times of each PD patient between assays. There was no correlation between the PMCA and RT‐QuICR assays for either measure. T50 values showed a weak correlation between the RT‐QuIC‐FG and RT‐QuICR assays. Pearson’s r and two‐tailed p‐values are reported, n = 105.
**Figure S2.** Correlation between UPDRS motor scores differs based on RBD status. Scatter plots of A, B) UPDRS part III and C, D) ΔUPDRS part III versus maximum fluorescence for A, C) RBD‐negative and B, D) RBD‐positive PD patients. ΔUPDRS part III is calculated from scores from visit 2 (off medication) – visit 1 (on medication) as a measure of PD medication efficacy. These measures inversely correlate with maximum fluorescence only in RBD‐negative patients. Pearson’s r and two‐tailed P‐values are reported, RBD‐negative group: n = 39; RBD‐positive group: n = 69.
**Table S1.** BioFIND patient demographic information.
**Table S2.** Correlation between T50 and maximum fluorescence and clinical and biochemical measures of PD patients.
