# Supplementary material for: A rapid α‐synuclein seed assay of Parkinson’s disease CSF panel shows high diagnostic accuracy
Source: Ann Clin Transl Neurol. 2020 Dec 29;8(2):374–84. doi: 10.1002/acn3.51280 (PMC7886040; doi:10.1002/acn3.51280)
Supplement: Supplementary file 1 — Figure S1. Comparison of RT‐QuICR, RT‐QuIC‐FG, and PMCA αSyn seeding assays. Scatter plots of A) maximum fluorescence in arbitrary units (au) and B) T50 times of each PD patient between assays. There was no correlation between the PMCA and RT‐QuICR assays for either measure. T50 values showed a weak correlation between the RT‐QuIC‐FG and RT‐QuICR assays. Pearson’s r and two‐tailed p‐values are reported, n = 105. Figure S2. Correlation between UPDRS motor scores differs based on RBD status. Scatter plots of A, B) UPDRS part III and C, D) ΔUPDRS part III versus maximum fluorescence for A, C) RBD‐negative and B, D) RBD‐positive PD patients. ΔUPDRS part III is calculated from scores from visit 2 (off medication) – visit 1 (on medication) as a measure of PD medication efficacy. These measures inversely correlate with maximum fluorescence only in RBD‐negative patients. Pearson’s r and two‐tailed P‐values are reported, RBD‐negative group: n = 39; RBD‐positive group: n = 69. Table S1. BioFIND patient demographic information. Table S2. Correlation between T50 and maximum fluorescence and clinical and biochemical measures of PD patients. [file ACN3-8-374-s001.docx]

Supplementary Information for:

**A rapid α-synuclein seed assay of Parkinson’s disease CSF panel shows high diagnostic accuracy**

Christina D. Orrù^1^^§^, Thong C. Ma^2§^, Andrew G. Hughson^1^, Bradley R. Groveman^1^, Ankit Srivastava^1^, Douglas Galasko^4^, Rachel Angers^5^, Patrick Downey^5^, Karen Crawford^6^, Samantha J. Hutten^7^, Un Jung Kang^2*^, Byron Caughey^1*^


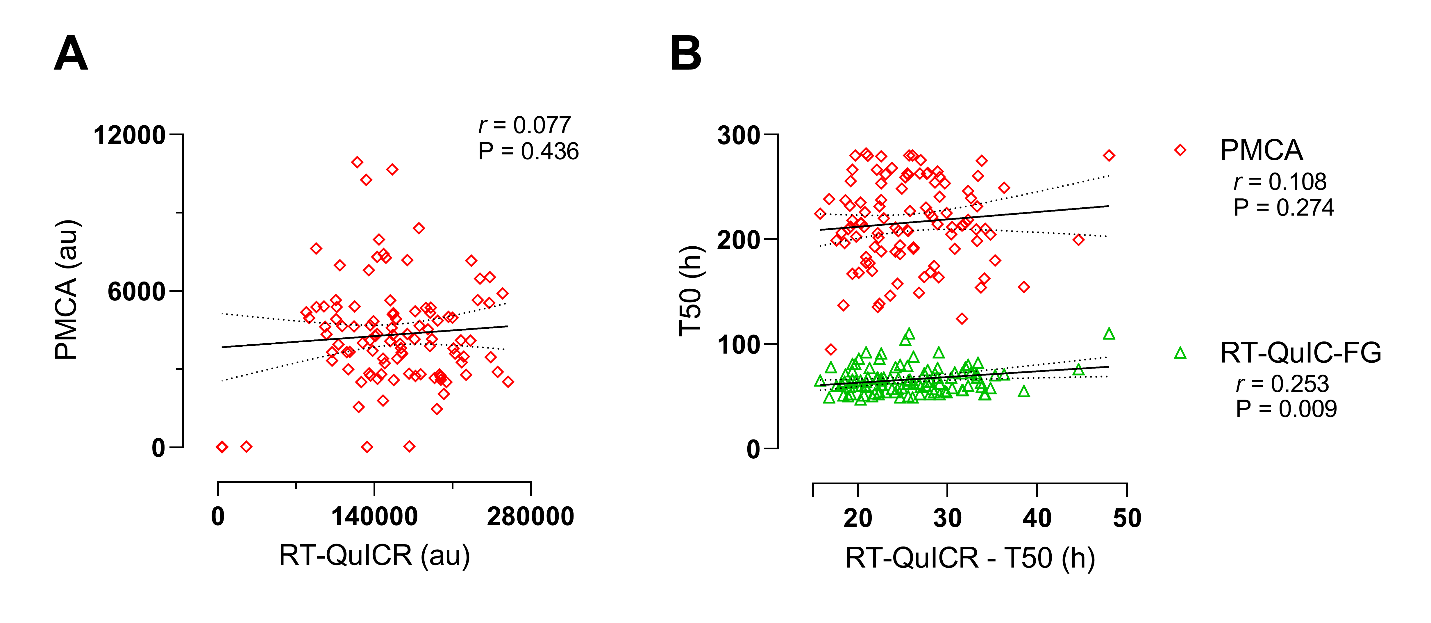
 **Supplementary Figure S1. Comparison of RT-QuICR, RT-QuIC-FG, and PMCA αSyn seeding assays.** Scatter plots of A) maximum fluorescence in arbitrary units (au) and B) T50 times of each PD patient between assays. There was no correlation between the PMCA and RT-QuICR assays for either measure. T50 values showed weak correlation between the RT-QuIC-FG and RT-QuICR assays. Pearson’s r and two-tailed p-values are reported, n=105.


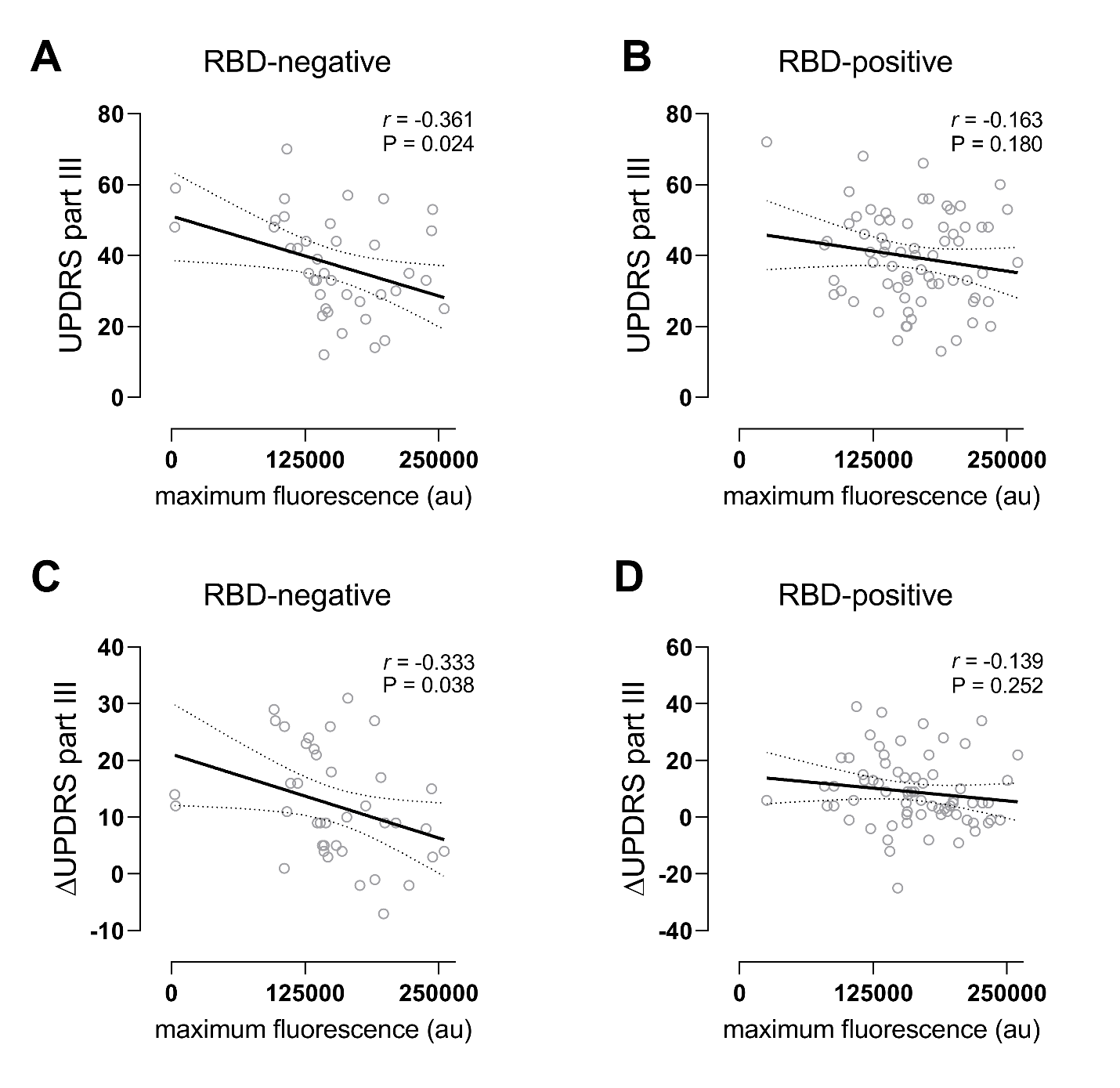
 **Supplementary Figure S2. Correlation between UPDRS motor scores differs based on RBD status.** Scatter plots of A, B) UPDRS part III and C, D) ΔUPDRS part III versus maximum fluorescence for A, C) RBD-negative and B, D) RBD-positive PD patients. ΔUPDRS part III is calculated from scores from visit 2 (off medication) – visit 1 (on medication) as a measure of PD medication efficacy. These measures inversely correlate with maximum fluorescence only in RBD-negative patients. Pearson’s r and two-tailed P-values are reported, RBD-negative group: n=39; RBD-positive group: n=69.

**Supplementary Table S1. BioFIND patient demographic information**

| Subjects | Total (N = 193) | PD (N = 108) | Control (N = 85) | P-value |
| --- | --- | --- | --- | --- |
| Male, N (%) | 105 (54.4%) | 65 (60.2%) | 40 (47.1%) | 0.0691 |
| Age at enrollment, years | 66 [53-84] | 68 [55-82] | 65 [53-84] | 0.0103 |
| UPDRS total | - | 59 [22-133] | - | - |
| UPDRS part III | 22 [0-72] | 39 [12-72] | 1 [0-14] | <0.0001 |
| H&Y Stage | 2 [0-4] | 2 [1-4] | 0 [0-0] | <0.0001 |
| Disease duration, years | - | 8 [4-19] | - | - |
| MoCA | 28 [19-30] | 27 [19-30] | 28 [23-30] | 0.0165 |

N = number of subjects; Continuous data are reported as median [range]

PD: Parkinson’s disease; Control: healthy individuals

UPDRS (Unified Parkinson’s Disease Rating Scale); MoCA (Montreal Cognitive Assessment);

H&Y stage (Hoehn and Yahr scale)

Subjects were off PD medication for assessment (during visit 2 for PD patients)

P-values are for PD vs. Control; Chi-square test for %male; Mann-Whitney test for continuous data

**Supplementary Table S2. Correlation between T50 and maximum fluorescence and clinical and biochemical measures of PD patients.**

| ***T50 vs:*** | | **r** | **R-squared** | **P-value** |
| --- | --- | --- | --- | --- |
|  | age | 0.102 | 0.010 | 0.294 |
|  | symptom duration | -0.136 | 0.018 | 0.161 |
|  | diagnosis duration | -0.150 | 0.022 | 0.122 |
|  | MOCA | 0.031 | 0.001 | 0.752 |
|  | H&Y – visit 1 (on) | 0.109 | 0.012 | 0.264 |
|  | H&Y – visit 2 (off) | 0.091 | 0.008 | 0.349 |
|  | UPDRS part III - visit 1 (on) | 0.014 | 0.000 | 0.883 |
|  | UPDRS part III - visit 2 (off) | -0.002 | 0.000 | 0.986 |
|  | UPDRS total - visit 1 (on) | -0.020 | 0.000 | 0.839 |
|  | UPDRS total - visit 2 (off) | -0.031 | 0.001 | 0.747 |
|  | ΔUPDRS part III (visit 1 – visit 2) | -0.020 | 0.000 | 0.840 |
|  | RBD question 6 | -0.253 | 0.064 | * 0.008 |
|  | RBD total | -0.200 | 0.040 | * 0.038 |
|  | CSF α-synuclein | 0.082 | 0.007 | 0.396 |
|  | CSF phospho-tau | 0.232 | 0.054 | * 0.016 |
|  | CSF total tau | 0.098 | 0.010 | 0.315 |
|  |  |  |  |  |
| ***Maximum fluorescence vs:*** | | **r** | **R-squared** | **P-value** |
|  | age | -0.105 | 0.011 | 0.279 |
|  | symptom duration | 0.052 | 0.003 | 0.590 |
|  | diagnosis duration | 0.023 | 0.001 | 0.811 |
|  | MOCA | -0.058 | 0.003 | 0.551 |
|  | H&Y – visit 1 (on) | 0.146 | 0.021 | 0.132 |
|  | H&Y – visit 2 (off) | 0.017 | 0.000 | 0.863 |
|  | UPDRS part III - visit 1 (on) | -0.047 | 0.002 | 0.629 |
|  | UPDRS part III - visit 2 (off) | -0.233 | 0.054 | * 0.015 |
|  | UPDRS total - visit 1 (on) | -0.027 | 0.001 | 0.781 |
|  | UPDRS total - visit 2 (off) | -0.147 | 0.022 | 0.128 |
|  | ΔUPDRS part III (visit 1 – visit 2) | -0.214 | 0.046 | * 0.026 |
|  | RBDQ question 6 | 0.022 | 0.000 | 0.825 |
|  | RBDQ total | 0.094 | 0.009 | 0.333 |
|  | CSF α-synuclein | -0.209 | 0.044 | * 0.030 |
|  | CSF phospho-tau | -0.055 | 0.003 | 0.575 |
|  | CSF total tau | -0.211 | 0.045 | * 0.028 |

Pearson’s r value and two-tailed P-value are reported, * P < 0.05.

Abbreviations as defined in previous tables or text.
